# Short-term balance consolidation relies on the primary motor cortex: a rTMS study

**DOI:** 10.1038/s41598-023-32065-x

**Published:** 2023-03-30

**Authors:** S. Egger, M. Wälchli, E. Rüeger, W. Taube

**Affiliations:** 1 Department of Neurosciences and Movement Science, University of Fribourg, Fribourg, Switzerland; 2 Medicine Section, Department of Neurosciences and Movement Sciences, Faculty of Science and Medicine, University of Fribourg, Bd de Pérolles 90, Office F440, 1700 Fribourg/Freiburg, Switzerland

**Keywords:** Consolidation, Motor cortex

## Abstract

Structural and functional adaptations occur in the primary motor cortex (M1) after only a few balance learning sessions. Nevertheless, the role of M1 in consolidating balance tasks remains to be discussed, as direct evidence is missing due to the fact that it is unclear whether adaptations in M1 are indeed the driving force for balance improvements or merely the consequence of improved balance. The aim of the present study was to investigate whether the primary motor cortex is involved in the learning and consolidation of balance tasks. Thirty participants were randomly allocated into a repetitive transcranial magnetic stimulation (rTMS) or sham-rTMS group. The experimental design included a single balance acquisition phase, followed by either 15 min of low-frequency rTMS (1 Hz at 115% of resting motor threshold to disrupt the involvement of M1) or sham-rTMS, and finally a retention test 24 h later. During the acquisition phase, no differences in balance improvements were observed between the two groups. However, significant differences between the rTMS and the sham-rTMS group were found from the end of the acquisition phase to the retention test. While the rTMS group had a performance loss, the sham-rTMS group displayed significant off-line gains (*p* = 0.001). For the first time, this finding may propose a causal relationship between the involvement of M1 and the acquisition and consolidation of a balance task.

## Introduction

The human brain is capable of learning numerous new motor tasks through practice. A crucial role in the learning process of motor tasks is attributed to the plasticity of the primary motor cortex (M1)^1–4^. However, M1 is assumed to be differently involved depending on the motor task and the motor learning paradigm. For instance, for a long time, balance control and balance learning were considered to rely mainly on spinal reflex circuits^5,6^ and subcortical structures (for reviews, see^7,8^) such as the brainstem, cerebellum and the basal ganglia. More recently, knowledge emerged that higher cortical centers—and especially M1—also adapt in response to balance learning (for reviews, see^9,10^). Studies using transcranial magnetic stimulation (TMS) demonstrated reduced excitability^11–13^ and increased short-interval intracortical inhibition (SICI) of M1^14,15^ after several weeks of balance training. Similarly, imaging methods indicated functional and structural adaptations in M1 after a few balance learning sessions^16–18^. In addition, EEG studies^9^ as well as behavioral studies (e.g. dual-task^19,20^ and motor interference paradigms^21^) indicated involvement of higher-order cognitive functions in balance control, in all likelihood involving M1^9^. Furthermore, cognitive impairment is known to increase the risk of losing balance, indicating a relation between cortical involvement and balance performance^22^. However, despite the high interest in balance control in general and the role of M1 in particular, the role of M1 in consolidating balance tasks is still not clear. The reason for this is that causal studies are missing and that it is a distinct possibility that adaptations in M1 might be a consequence of improved balance (e.g. less postural sway) rather than the actual driving force for improved balance coordination.

One safe, non-invasive technique to investigate the impact of M1 in learning processes more directly is to impair the consolidation with the application of repetitive TMS (rTMS). Low-frequency rTMS over M1 is usually assumed to cause an inhibitory effect on motor cortical excitability and thus, interferes with cortical processes^23^. If rTMS disrupts such functional brain processes, it is often described as reversible virtual lesion^24^, and associated with a negative influence on the consolidation of recently learned motor tasks. It was already shown that rTMS over M1 can impair the consolidation of ballistic finger^25,26^, ankle^27^ and wrist motor tasks^28^ but not the consolidation of a single digit force field task^25^. Thus, depending on the motor task, M1 is thought to be differently involved and rTMS seems an appropriate method to disrupt the involvement of M1 and impair motor memory consolidation.

For the current study, we hypothesized that if M1 is (at least partly) responsible for the adaptations in balance control, application of rTMS should impair consolidation of balance tasks. In contrast, if the reported changes in M1 (e.g. reduced excitability of M1 or enhanced SICI) are only the consequence of improved balance control (e.g. less postural sway) but are not the driving force, disruption of M1 should not impair consolidation of balance tasks. For this purpose, two groups were tested, one receiving rTMS and the other sham rTMS over M1 after having performed a balance learning task. The results showed an improved balance performance in the retention test in participants who received sham-rTMS and decreased performance in participants with the application of rTMS. Thus, our study proposes the direct involvement of M1 in the early phase of learning and consolidating a balance task.

## Results

**Figure 1:**
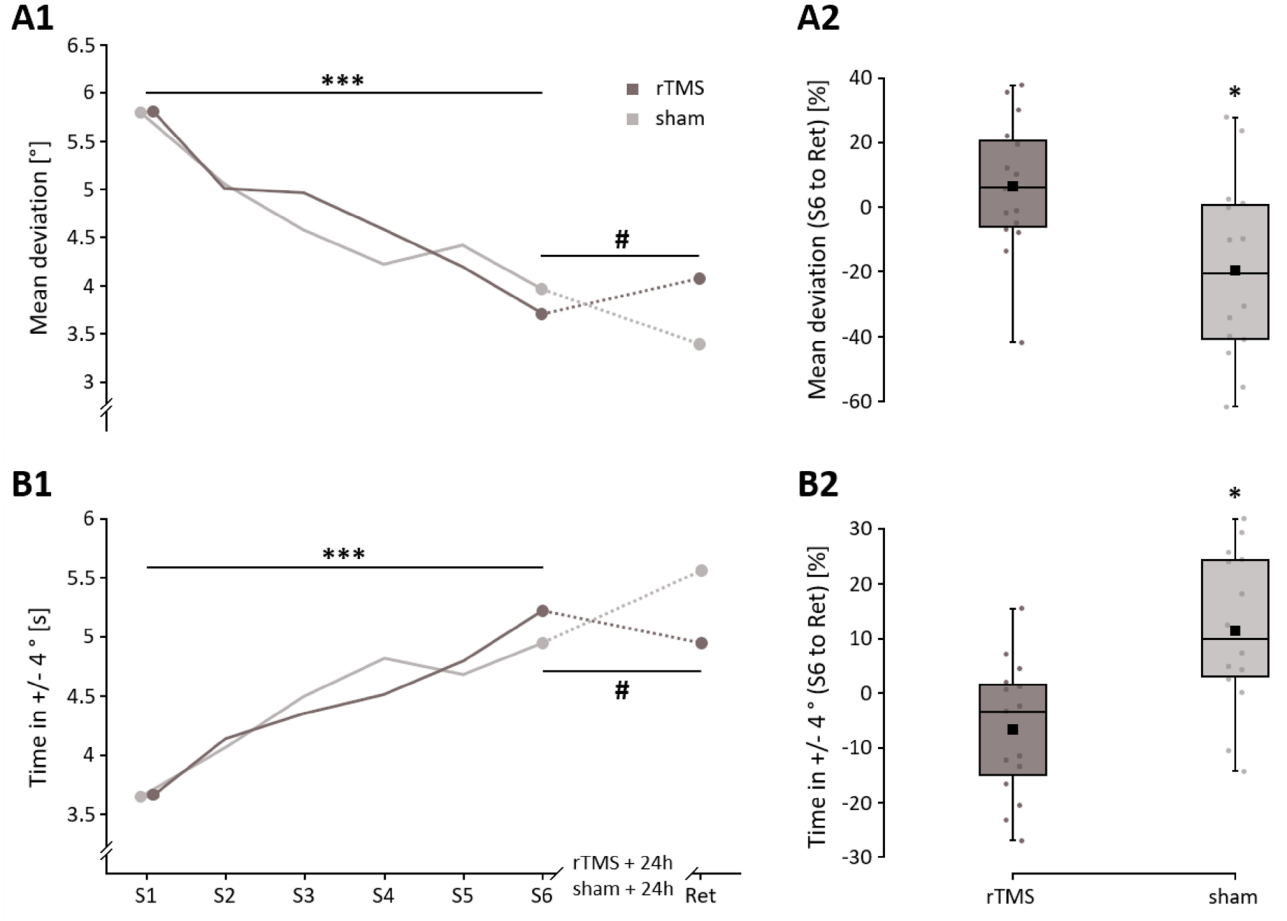
Raw performance on the rocker-board (**A1**,**B1**) and percentage changes (consolidation) from S6 to Ret (**A2**,**B2**) for the parameters mean deviation and Time in ± 4°. Values lower than zero percent in (**A2**) indicate that performance has improved. *rTMS* repetitive transcranial magnetic stimulation. *Ret* retention. ***p < 0.001; *p < 0.05. ^#^Significant interaction (*p* < 0.05).

Both balance parameters (i.e. ‘mean deviation’ and ‘time in ± 4°’) indicate that the postural performance on the rocker-board significantly improved during the acquisition phase from S1 to S6 (‘mean deviation’: *F*_1,27_ = 81.613, *p* < 0.001, *n*^2^*p* = 0.751; ‘time in ± 4°’: *F*_1,27_ = 67.099, *p* < 0.001, *n*^2^*p* = 0.713) independent of the intervention group (rTMS vs. sham-rTMS ‘mean deviation’: *F*_1,27_ = 0.059, *p* = 0.810, *n*^2^*p* = 0.002; ‘time in ± 4°’: *F*_1,27_ = 0.147, *p* = 0.704, *n*^2^*p* = 0.005; see Fig. 1). This is further supported by the comparable percentage improvements for the rTMS (‘mean deviation’: − 63.42% and ‘time in ± 4°’: 28.97%) and sham-rTMS (‘mean deviation’: − 55.82% and ‘time in ± 4°’: 25.03%) group. Consequently, TIME*GROUP interaction was not significant for both parameters (‘mean deviation’: *F*_1,27_ = 0.326, *p* = 0.573, *n*^2^*p* = 0.012; ‘time in ± 4°’: *F*_1,27_ = 0.594, *p* = 0.488, *n*^2^*p* = 0.022), which indicates that both groups had a similar performance improvement from S1 to S6.

However, the significant TIME*GROUP interaction of the ANOVA revealed differences in consolidation (i.e. from S6 to Ret) between the rTMS and sham-rTMS group (‘mean deviation’: *F*_1,27_ = 10.775, *p* = 0.003, *n*^2^*p* = 0.285; ‘time in ± 4°: *F*_1,27_ = 10.183, *p* = 0.004, *n*^2^*p* = 0.274, see Fig. 1). Corrected separate post-hoc tests revealed a significant improvement for both parameters from S6 to Ret for the sham-rTMS group (‘mean deviation’: *t*_27_ = 2.802, p* = 0.018; ‘time in ± 4°’: *t*_27_ = − 3.103, p* = 0.008) and a non-significant decrease for the rTMS group (‘mean deviation’: *t*_27_ = − 1.824, p* = 0.158; ‘time in ± 4°’: *t*_27_ = 1.381, p* = 0.358). None of the other factors (i.e. TIME, GROUP,) of the ANOVA revealed a significant main effect. The ANOVA results of the raw values are confirmed by the percentage changes of the two balance parameters from S6 to Ret. The sham-rTMS group achieved significantly better consolidation values than the rTMS group (‘mean deviation’: *t*_27_ = 2.780, *p* = 0.010, *d* = 1.033; rTMS: 6.35% vs. sham-rTMS: − 19.56%; ‘time in ± 4°’: *t*_27_ = − 3.599, *p* = 0.001, *d* = − 1.337; rTMS: − 6.57% vs. sham-rTMS: 11.51%).

## Discussion

The present study proposes for the first time a causal relationship between the involvement of M1 and the early learning and consolidation of a balance task. This can be concluded as the rTMS group demonstrated a significantly worse consolidation (i.e. numerical loss of performance from the end of the acquisition phase to the start of the retention test) of the balance task in contrast to the sham-rTMS group (which showed a significant increase in performance). Previous studies had demonstrated that higher cortical centers (especially M1) adapt in response to balance learning^9,10,16–18^. However, these studies could not clarify whether adaptations in M1 are solely the consequence of less postural sway or whether adaptations in M1 were indeed the driving force for balance skill enhancements. It was therefore the aim of the present study to interfere with the functionality of M1 in order to clarify whether M1 is directly involved in learning and consolidating balance tasks. The underlying mechanism of how rTMS interferes with the consolidation of motor tasks is still unclear. However, the applied rTMS protocol (900 pulses at 1 Hz) normally induces a kind of long-term depression^29^, which probably impairs synaptic efficacy in M1 and thus, interferes with the consolidation of motor tasks that rely on M1. In this way, the involvement of M1 in skill learning has been demonstrated for several simple motor tasks such as ballistic finger^25,26^, ankle^27^ and wrist motor tasks^28^. However, rTMS does not interfere with the consolidation of all motor tasks. Thus, it is assumed that tasks that do not rely very strongly on M1 are not affected by rTMS as was shown for some force field tasks^25^. It is further supposed that the higher the task complexity, the more different cortical centers are active and thus, consolidation might be more distributive, lessening the disruptive influence of rTMS on M1^25^. At first glance, our current results do not support the latter assumption as balancing can be considered to be highly complex not only from the motor execution side, as many different muscles and joints have to be coordinated, but also from the aspect of multiple sensory integration. However, when comparing the interfering effect of rTMS (performance decrease of 6.4%) with the interfering effect of learning a second balance task (performance decrease of 20.6%; see Egger, et al.^21^, it can be seen that rTMS caused less interference than the learning of a complex postural task. Noteworthy, the same balance device and the same number of repetitions were used in both studies. This might indeed suggest that other supraspinal areas may be responsible for consolidation of the balance task that are not targeted by rTMS over M1. This assumption is in line with previous research showing that many different areas of the central nervous system are involved in balance learning ^[^for a review see^10^]. In addition, the difficulty of the task may lead to varying amounts of activity in different cortical and subcortical centers^30^. However, only challenging balance tasks revealed significant activity in M1 in an fMRI study in which participants performed mental simulation of different postural tasks^30^. In the present study, the difficulty level was adjusted for each participant individually to ensure that the task was challenging. The present results can therefore not be generalized for the learning of easier balance tasks that might involve less contribution of M1. Furthermore, it has to be noted that the present study did not involve perturbation of posture. Thus, the current task, despite being more challenging, might be similarly controlled as ‘simple upright stance’^31,32^ in a feedforward manner. This means that the consequences of muscular activations onto posture could be well anticipated. Such an anticipatory postural control may rely on high contributions of cortical centers and more specifically M1. The involvement of M1 when learning to adapt to postural perturbations may be different and the results of the present study can therefore not be used to infer the role of M1 in learning reactive balance tasks.

When balance skills are learned over a longer-time period, the involvement of cortical centers seems to decrease^12,13^ and it is assumed that balance skills then depend more on subcortical regions^11^. If this shift from cortical centers to subcortical regions is indeed occurring, interference by rTMS should have less and less influence on the consolidation of a balance task with progressive training. This temporal aspect was investigated for a maximal voluntary force task^33^. The results demonstrated that rTMS diminished the increase in strength even over 10 sessions. Whether balance tasks are susceptible to interference by rTMS over a similar time period remains to be investigated by further studies. However, the present results indicate that for the initial acquisition phase of a balance task (i.e. for a single training session), involvement of M1 is essential for short-term learning and consolidation.

Despite the meaningful results, potential limitations of the study must be addressed. Since suprathreshold rTMS was applied, the intensity of the stimulation was sufficient to contract the target muscle and therefore, also led to re-afferent activity. For this reason, it cannot be excluded that not only the disruption of M1 was responsible for the impaired consolidation but also the afferent input from the target muscle^27^. Furthermore, it is a distinct possibility that the stimulation over M1 not only had an influence on M1 but may have also influenced other remote brain structures in a direct and/or indirect way^24^. This should be considered especially since it is already known that not only different subcortical brain structures^7,8^, such as the brainstem, cerebellum or basal ganglia, but also other higher cortical areas^9,10^, like the M1, dorsolateral prefrontal cortex or supplementary motor cortex contribute to balance control. Thus, it is strongly assumed that these brain structures work together in an orchestrated manner to integrate sensory information and adapt the motor output^34,35^.

## Methods

### Participants

Thirty young and healthy volunteers participated in the study. They were naïve to the balance task and were not permitted to participate if they met any exclusion criteria for safety TMS application^36^. Participants were randomly allocated into the rTMS or sham-rTMS group without knowing their group affiliation. Before starting the experiment, participants gave written informed consent. The whole procedure was in accordance with the declaration of Helsinki and was allowed by the local ethics committee (Commission cantonale d'éthique de la recherche sur l'être humain (CER-VD); ID: 2022–01526). One female participant was excluded from the study because she showed no changes in performance during the initial balance acquisition phase, which took place before the application of rTMS/sham rTMS. In total, 29 participants (rTMS: *n* = 15, 6 women, 24.0 ± 2.8 years, 66.6 ± 7.4 kg, 1.74 ± 0.07 m; sham rTMS: *n* = 14, 4 women, 22.9 ± 3.2 years, 68.2 ± 10.3 kg, 1.76 ± 0.08 m) were considered for data analysis.

### Experimental design

**Figure 2:**
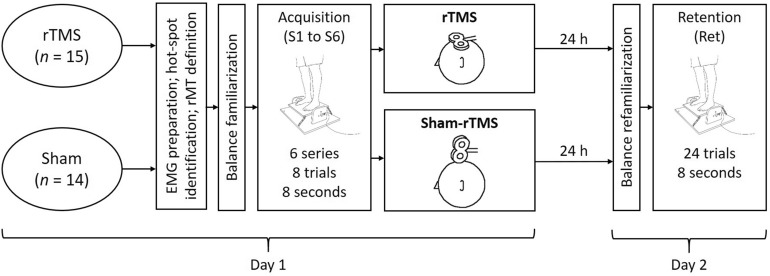
Summary of the experimental design. *rTMS* repetitive transcranial magnetic stimulation, *n* number of participants, *S1* series 1. *EMG* electromyography, *rMT* resting motor threshold.

The experimental design (Fig. 2) included a single balance learning acquisition phase, followed by either rTMS or sham-rTMS, and finally a retention test 24 h later. Before the acquisition phase, the participants were prepared for electromyographic (EMG) measurements. Subsequently, the optimal position (hot-spot) to stimulate the soleus muscle (SOL) with TMS was identified, marked and the corresponding resting motor threshold (rMT) was determined for each participant individually. For the acquisition phase, participants completed 6 series (S1 to S6) with 8 trials per series on the balance device after a brief familiarization (6 trials). As fast as possible (approximately 3 min) after the balance acquisition phase, participants received either rTMS (1 Hz at 115% of rMT) or sham-rTMS (1 Hz stimulations with the coil rotated by 90°) for 15 min (i.e. 900 stimulations). After the TMS procedures (rTMS or sham-rTMS), we alerted the participants not to follow any physical activities until the retention test. Subsequently, they were free to leave the laboratory. The following day (24 h later), participants performed a retention test (Ret), which included a short re-familiarization (6 trials, identical to the familiarization in the acquisition phase) and 24 trials on the balance device.

### Balance task

Participants stood shoulder width on the platform of a rocker-board that could rotate forward and backward to a maximum of 20° around a fixed axis and were asked to keep the platform as quiet and horizontal as possible during each of the 48 trials. After each trial, which lasted 8 s, participants received offline feedback on their performance on a screen, standardizing the pause length to 30 s. Feedback reflected the platform position relative to the horizontal alignment and the corresponding mean deviation [°/8 s] from the horizontal line as a single value. Participants were encouraged to minimize this value as much as possible throughout the experiment. Between the series, participants had a pause of 60 s. Aborted trials (leaving the device or holding on) were repeated. A detailed description of the balance device (i.e. rocker-board) was previously provided^21^.

### Repetitive transcranial magnetic stimulation (rTMS)

Repetitive magnetic stimuli were delivered for 15 min at a rate of 1 Hz and with an intensity of 115% rMT to the contralateral (e.g. left) hemisphere of M1 via an air-cooled figure of eight coil (MCF-B65, Tonica Elektronik A/S, Farum, Denmark) connected to a Magstim stimulator (MagPro X100 with MagOption, MagVenture A/S, Farum, Denmark). The hot-spot to elicit motor evoked potentials (MEPs) in the SOL was marked (with color directly on the scalp) and the coil was fixed tangentially to the scalp by a mechanical mount. To facilitate the mounting, the coil was aligned straight over the head and the current flow had an anterior–posterior direction. RMT was afterwards defined in a seated position as the intensity that triggered MEPs peak-to-peak amplitudes greater than 50 µV in at least 5 out of 10 trials. For the sham-rTMS group the procedure was the same except that the coil was placed orthogonally to the scalp. Thus, the side of one winding touched the scalp but the TMS pulses were not able to stimulate the brain. All TMS measurements were conducted in compliance with safety recommendations^36^ and were visually monitored. That is, in both rTMS and sham rTMS, the presence or absence of MEPs was visually verified and guaranteed that rTMS evoked MEPs of around 0.3 mV and sham-rTMS did not evoke a muscular response.

### Electromyography (EMG)

Bipolar EMG from the right SOL was recorded with a custom-made system (EISA, University of Freiburg, Germany). The SOL was selected because this muscle is one of the prime movers in this particular task in the anterior–posterior direction. The skin was prepared (shaved, abraded and sanitized) before the surface electrodes (Ag/AgCI, Ambu Blue Sensor P, Ballerup, Denmark) were firmly placed over the muscle belly and the tibia shaft (reference electrode). All electrodes were attached according to SENIAM guidelines^37^. EMG signals were sampled (2 kHz), amplified (× 1000) and band-pass filtered (10–1000 Hz) for online recording (Imago Record, Pfitec, Endingen, Germany) and further offline analysis (R2017b, The MathWorks, Inc., Natick, MA, USA).

### Data processing

Matlab was used for offline data processing. For each series (S1 to S6) of the acquisition phase, the mean of the 8 trials was calculated. For the Ret phase, the mean value of all 3 series was used. Mean values were calculated (a) for the mean deviation [°/8 s], which participants received as feedback after each trial, and (b) for the duration [s] participants balanced in ± 4° to the horizontal alignment during each trial.

### Statistical analysis

All data has been verified to be normally distributed. Statistical tests were performed with SPSS (IBM SPSS Statistics 23, IBM Corporation, Armonk, USA). Performance development on the first day (i.e. acquisition phase) was tested with a repeated mixed design analysis of variance (ANOVA) with TIME (S1; S6) as within and GROUP (rTMS; sham-rTMS) as between factor. Consolidation from S6 to Ret was tested by ANOVA with TIME (S6; Ret) as within and GROUP (rTMS; sham-rTMS) as between factor. In order to further investigate the differential consolidation effects of rTMS and sham-rTMS, percentage changes were additionally illustrated and statistically analyzed using an independent samples *t*-test. The significance level was defined at *p* < 0.05 and effect sizes of the variance analyses are indicated by the partial eta square (*η*^2^*p*; small: 0.02; medium: 0.13; large: 0.26). For the significant *F*-values of the variance analyses, twofold corrected separate post-hoc tests (p*) were calculated for the development from S6 to Ret for both groups independently as an index of the consolidation. Effect sizes of the independent *t*-tests are reported by Cohens-*d* (*d*; small: 0.2; medium: 0.5; large: 0.8).

## Data Availability

The raw data that led to this article will be made available by the corresponding author to any qualified researcher.
